# Protective Effects of MitoTEMPO on Nonalcoholic Fatty Liver Disease via Regulating Myeloid-Derived Suppressor Cells and Inflammation in Mice

**DOI:** 10.1155/2020/9329427

**Published:** 2020-07-30

**Authors:** Jiayuan Wu, Li Zheng, Juanfen Mo, Xiujuan Yao, Chenliang Fan, Yi Bao

**Affiliations:** ^1^The Key Laboratory, The Second Affiliated Hospital of Jiaxing University, Jiaxing, Zhejiang 314000, China; ^2^Department of Pathology, The Second Affiliated Hospital of Jiaxing University, Jiaxing, Zhejiang 314000, China; ^3^Clinical Laboratory, The Second Affiliated Hospital of Jiaxing University, Jiaxing, Zhejiang 314000, China

## Abstract

MitoTEMPO, a mitochondrial antioxidant, has protective effects on liver-related diseases. However, the role of MitoTEMPO on nonalcoholic fatty liver disease (NAFLD) and its possible mechanisms are largely unknown. Here, we investigated the effects of MitoTEMPO on NAFLD using high fat diet- (HFD-) induced obese mice as animal models. MitoTEMPO was intraperitoneally injected into HFD mice. Liver morphological changes were observed by H&E and Oil Red O staining, and the frequency of MDSCs in peripheral blood was analyzed by flow cytometry. Moreover, real-time quantitative PCR, western blot, and immunohistochemistry were conducted to detect the mRNA and protein expressions in the liver tissues. The results showed that the hepatic steatosis in liver tissues of HFD mice injected with MitoTEMPO was significantly ameliorated. Additionally, MitoTEMPO reduced the frequency of CD11b^+^Gr-1^+^ MDSCs in peripheral circulation and decreased Gr-1^+^ cell accumulation in the livers. Further studies demonstrated that MitoTEMPO administration suppressed the mRNA and protein expressions of MDSC-associated proinflammatory mediators, such as monocyte chemoattractant protein-1 (MCP-1), S100 calcium-binding protein A8 (S100A8), and S100 calcium-binding protein A9 (S100A9). Our results suggest that MitoTEMPO appears to be a potential chemical compound affecting certain immune cells and further ameliorates inflammation in obese-associated NAFLD.

## 1. Introduction

Nonalcoholic fatty liver disease (NAFLD) is a common pandemic disease [1], which is affecting about 1/3 of the adult population in developed countries [2, 3]. According to disease severity, NAFLD could progress from steatosis to nonalcoholic steatohepatitis (NASH), fibrosis, and cirrhosis and finally develop to hepatocellular carcinoma (HCC) [4].

Accumulating evidence suggests that high fat diet- (HFD-) induced obesity contributes to the development of NAFLD [5, 6]. Moreover, NAFLD induced by obesity closely associates with immune dysfunction in the liver [7] and reflects a generalized chronic proinflammatory state [8, 9]. In the liver of obesity, the resident immune cells such as macrophages (Kupffer cells) and dendritic cells produce proinflammatory chemokines and cytokines, including monocyte chemoattractant protein-1 (MCP-1), interleukin-1*β* (IL-1*β*), and tumor necrosis factor-*α* (TNF-*α*) in response to hepatocellular damage [10–12]. MCP-1 further mediates the infiltration of chronic inflammatory cells such as macrophages and monocytes to the liver, resulting in amplification of inflammation in the liver microenvironment [11].

In addition to obesity, oxidative stress appears to be another risk factor aggravating NAFLD [13]. Therefore, antioxidant administration may be an effective way to prevent and ameliorate NAFLD. MitoTEMPO is a new cell permeable mitochondrial antioxidant with the functions of eliminating mitochondrial superoxide and preserving mitochondrial membrane potential [14]. It has been reported that MitoTEMPO could prevent N-nitrosodiethylamine-induced hepatocarcinogenesis in mice [15]. In addition, using a methionine-choline-deficient (MCD) diet-induced NAFLD model, Ma et al. found that MitoTEMPO blockaded reactive oxygen species (ROS) production, reversed NAFLD-induced intrahepatic CD4^+^ T lymphocyte decrease, and finally delayed NAFLD-promoted hepatocarcinogenesis [16], indicating the potential therapeutic effects of MitoTEMPO on NAFLD. However, the mechanism by which MitoTEMPO ameliorates NAFLD remains unclear. Besides, the pathogenesis is different between humans and the MCD-induced mouse NAFLD model. Therefore, it is necessary to explore the role of MitoTEMPO on immunomodulatory in high fat diet-induced NAFLD, which is much closer to human NAFLD pathogenesis.

Here, we explored immune cell MDSCs and inflammatory activities in obesity-associated NAFLD and further investigated the protective effect of MitoTEMPO on alleviating the severity of NAFLD via regulating immune response in the liver tissues.

## 2. Materials and Methods

### 2.1. Animal Experiments

Thirty male healthy C57BL/6J mice (5 weeks old, each weighing 18-22 g) were obtained from Shanghai Sippr-BK Laboratory Animal Co., Ltd. (Certification No. SCXK (Hu) 2013-0016) and housed in standard laboratory conditions. After feeding one week, mice were divided randomly into three groups: a normal diet group (lean, *n* = 10), a high fat diet group (HFD, *n* = 10), and a HFD+MitoTEMPO group (HFD+Mito, *n* = 10). For drug administration, mice were intraperitoneally injected with 5 mg kg^−1^ MitoTEMPO (Sigma, USA) 5 times at the 6^th^, 8^th^, 10^th^, 12^th^, and 14^th^ weeks [17–19]. Mice were euthanized at the end of the 14^th^ week. For weight detection, mice were weighed every week. For flow analysis, fresh whole blood samples were collected before mice were killed. For morphological evaluation and RNA and protein extraction, each mouse liver was sectioned and one-half was stored in liquid nitrogen immediately, while the other was fixed in 10% neutral buffered formalin. All experiment procedures were used according to the institutional animal care guidelines. The subject was authorized by the Medical Ethical Committee of the Second Affiliated Hospital of Jiaxing University.

### 2.2. Hematoxylin and Eosin Staining

To evaluate liver morphological change in each group, 10% neutral buffered formalin-fixed liver tissues were embedded into paraffin. Then, tissues were cut into 4 *μ*m sections to conduct hematoxylin and eosin (H&E) (Servicebio, China) staining according to a standard method [20].

### 2.3. Oil Red O Staining

To detect lipid droplets in mice liver, frozen sections (10 *μ*m) were stained with Oil Red O reagent (Servicebio, China) based on a published procedure [21]. All the staining results were observed under a light microscope (Zeiss, Germany).

### 2.4. Flow Cytometry

CD11b^+^Gr-1^+^ MDSCs in peripheral blood were analyzed by flow cytometry using FITC-labeled CD11b (BD Biosciences, USA) and PE-labeled Gr-1 (BD Biosciences, USA) fluorochrome-conjugated monoclonal antibodies. Briefly, 50 *μ*L of fresh whole blood was incubated with 5 *μ*L of each antibody in the dark for 20 min at 4°C. Then, 1 mL red blood cell lysis buffer was added to each incubated sample for 10 min. Cells were cleaned by PBS and resuspended in 300 *μ*L PBS and finally tested by BD FACSCanto II machine with BD FACSDiva v6.1.3 software (BD Biosciences, USA).

### 2.5. RNA Extraction and Real-Time Quantitative PCR

Mice liver RNA was extracted using a TRIzol reagent (Invitrogen, USA) in light of the manufacturer's procedure. RNA was quantified and reversed transcribed into a cDNA-utilizing RT Master Mix reagent (Takara, Japan). Then, PCR amplification was performed in an ABI StepOnePlus machine with SYBR fluorescent dye (Takara, Japan). The primer sequences of *MCP-1*, *S100A8*, *S100A9*, *tissue inhibitor of matrix metalloproteinase 1* (*TIMP1*), *Collagen-I*, and *alpha-smooth muscle actin* (*α-SMA*) are listed in Table 1. Each mRNA level was normalized to the *GAPDH* gene expression. Relative mRNA expression was calculated by the 2^-*ΔΔ*Ct^ method.

### 2.6. Immunoblot Analysis

Mice liver lysates were extracted for immunoblot analysis as previously described [22]. For primary antibody incubation, *α*-SMA (1 : 500, Proteintech, USA), S100A8 (1 : 500, R&D, USA), S100A9 (1 : 1000, Abcam, UK), MCP-1 (1 : 1000, Abcam, UK), and *β*-actin (EarthOX, USA) were selected. For secondary antibody incubation, goat anti-mouse IgG (H+L) (Jackson ImmunoResearch, USA), goat anti-rabbit IgG (H+L) (Jackson ImmunoResearch, USA), and rabbit anti-goat IgG-HRP (cwbiotech, China) were used.

### 2.7. Immunohistochemical Analysis

Mouse Gr-1 (Servicebio, China) protein expression in the paraffin-embedded sections of liver tissues was detected by immunohistochemistry. In brief, paraffin-embedded liver tissues were cut into 5 *μ*m slides. After deparaffinizing and permeabilizing, antigen retrieval was conducted by EDTA buffer. 3% H_2_O_2_ was used to block endogenous peroxidase, and 10% goat serum was utilized to suppress the nonspecific bindings. Sections were incubated with diluted Gr-1 primary antibody overnight at 4°C. After washing in PBST, sections were incubated with HRP-labeled goat anti-rabbit IgG (Servicebio, China) for 1 h. The chromogenic reaction solution (DAKO, China) and counter-staining was performed with Harris hematoxylin for 3 min.

### 2.8. Statistical Analysis

The quantification analysis of immunoblot was conducted with ImageJ v1.8.0 software (National Institutes of Health, Germany). Data were analyzed using GraphPad Prism 6.0 software (GraphPad Software Inc., USA). All numerical calculations were presented as mean ± SEM. Unpaired *t*-test was used to evaluate the statistical difference between two groups. A value of *P* < 0.05 was considered as the criterion of statistical significance.

## 3. Results

### 3.1. MitoTEMPO Did Not Reduce HFD-Induced Body Weight Gain

**Figure 1:**
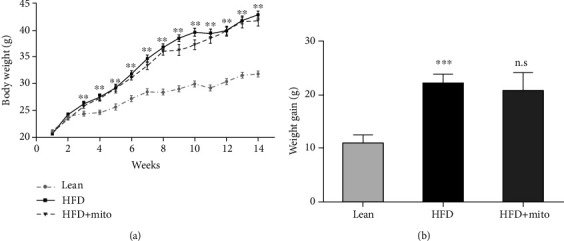
Effect of MitoTEMPO on body weight. (a) The body weight and (b) body weight gain in the lean, HFD, and HFD+Mito groups (*n* = 10, each group). HFD vs. lean: ^∗∗^*P* < 0.01, ^∗∗∗^*P* < 0.001, HFD+Mito vs. HFD: n.s: no significant difference, unpaired *t*-test.

The average body weight of the HFD group significantly increased compared with that of the lean group from the third week (*P* < 0.01) (Figure 1(a)). Notably, MitoTEMPO administration at the 6^th^, 8^th^, 10^th^, 12^th^, and 14^th^ weeks did not effectively reduce the body weight compared with the HFD group (*P* > 0.05) (Figure 1(a)). From the first week to the experimental end points, the average body weight gain in the HFD group (22.19 g ± 0.53 g) was significantly different compared with that in the lean group (10.89 g ± 0.51 g) (*P* < 0.01), whereas there was no statistical difference between the HFD+Mito (22.82 g ± 1.09 g) and the HFD group (22.19 g ± 0.53 g) (*P* > 0.05) (Figure 1(b)).

### 3.2. MitoTEMPO Ameliorated HFD-Induced Hepatic Steatosis and Decreased Lipid Accumulations

**Figure 2:**
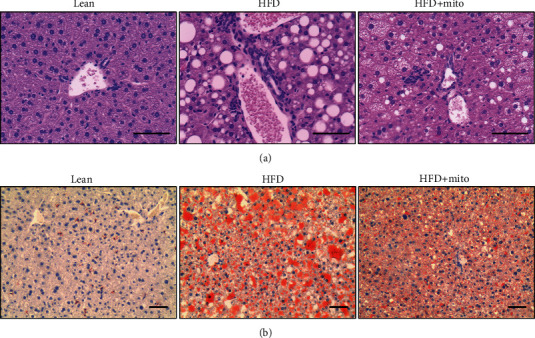
Morphological changes of liver tissues in mice. (a) Representative H&E staining of paraffin-embedded livers from the lean (left), HFD (middle), and HFD+Mito (right) groups. Scale bar, 100 *μ*m. (b) Representative Oil Red O staining in frozen sections of liver tissues from the lean (left), HFD (middle), and HFD+Mito (right) groups. Scale bar, 100 *μ*m.

Next, we evaluated the effects of MitoTEMPO on changes of high fat diet-induced hepatic steatosis and liver morphology. H&E staining revealed a manifesting appearance of hepatic steatosis, along with accumulation of vacuoles in the HFD group compared with the lean controls (Figure 2(a)). In addition, the number of infiltrated lymphocyte around portal vein areas in the liver tissues of the HFD group was also increased. Interestingly, MitoTEMPO-administered mice exhibited a remarkable reduction in vacuoles and lymphocyte infiltration compared with the HFD group (Figure 2(a)).

Oil Red O staining demonstrated that there were few lipid droplets in the lean group. In contrast, the HFD group mice showed severe and abundant lipid droplets in the hepatocytes. In contrast, MitoTEMPO administration markedly decreased the size and number of hepatic lipid droplets (Figure 2(b)).

### 3.3. MitoTEMPO Decreased HFD-Induced MDSCs in Peripheral Circulation and Liver Tissues

**Figure 3:**
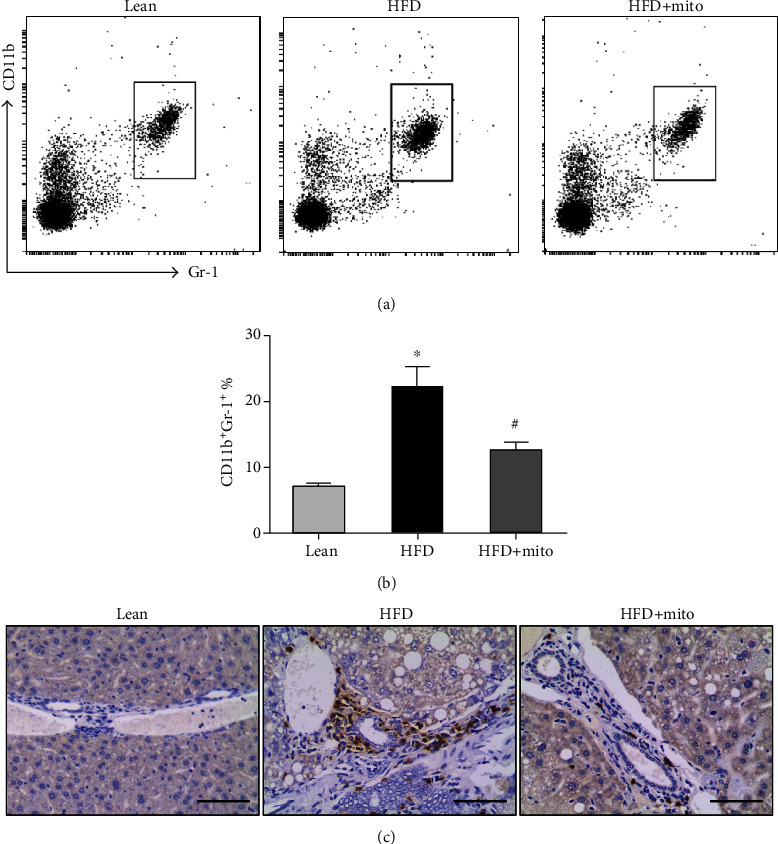
The frequency of CD11b^+^Gr-1^+^ MDSCs in mice. (a) Flow cytometry analysis of CD11b^+^Gr-1^+^ MDSCs in peripheral blood of the lean (left), HFD (middle), and HFD+Mito (right) groups. (b) Representative quantification of CD11b^+^Gr-1^+^ MDSCs in the three groups. Data are represented as the mean ± SEM. HFD vs. lean: ^∗^*P* < 0.05, HFD+Mito vs. HFD: n.s: no significant difference, unpaired *t*-test. (c) Gr-1 immunohistochemistry images in the lean (left), HFD (middle), and HFD+Mito (right) groups. Scale bar, 100 *μ*m.

Next, we investigated the effect of MitoTEMPO on immune activity in peripheral circulation and local tissue of mice. Flow cytometry was used to explore the frequency of CD11b^+^Gr-1^+^ MDSCs in peripheral circulation of mice (Figures 3(a) and 3(b)). The average percentage of MDSCs in the peripheral blood of the HFD group (22.20% ± 2.99%) was approximately 3-folds more than that of the lean group (7.00% ± 0.48%) (*P* < 0.05) (Figure 3(b)). In contrast, treatment with MitoTEMPO resulted in a 1.8-fold decrease in the percentage of CD11b^+^Gr-1^+^ MDSCs (12.52% ± 1.22%) compared with the HFD group (*P* < 0.05) (Figure 3(b)).

To further explore the recruitment of MDSCs to the liver tissues, we conducted immunohistochemistry to detect the liver-infiltrated Gr-1^+^ cells. Consistent with the flow cytometry data, there is only a small number of Gr-1^+^ cells around the hepatic portal vein area in the liver tissues of the lean group (Figure 3(c)). The number of Gr-1^+^ cells was obviously increased in the liver tissues of the HFD group (Figure 3(c)). Compared with the HFD group, the HFD+Mito group showed a reduction in the number of liver-infiltrated Gr-1^+^ cells (Figure 3(c)).

### 3.4. MitoTEMPO Inhibited HFD-Induced Inflammatory Response

**Figure 4:**
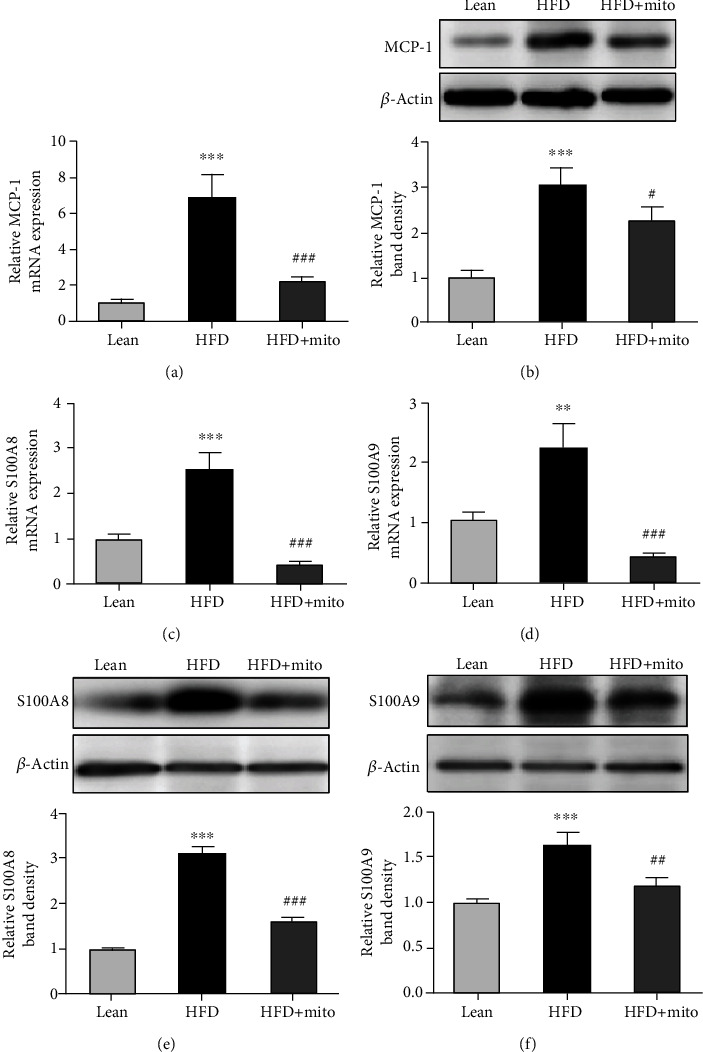
The mRNA and protein levels of liver chronic inflammatory response in mice. (a) The mRNA level of *MCP-1* by qRT-PCR assay. mRNA expression was normalized to *GAPDH* expression and shown as fold change (2^-*ΔΔ*Ct^). (b) The protein level of MCP-1 using immunoblot. The quantification of the intensity of MCP-1 relative to *β*-actin is shown below. (c, d) The mRNA levels of *S100A8* (c) and *S100A9* (d) in the liver tissues of each group were measured by qRT-PCR assay. (e, f) Western blot analysis of S100A8 (e) and S100A9 (f) protein expressions in the liver tissues of each group. Relative band density is shown in the bottom. Data are represented as the mean ± SEM. HFD vs. lean: ^∗∗^*P* < 0.01, ^∗∗∗^*P* < 0.001, HFD+Mito vs. HFD: ^###^*P* < 0.001, unpaired *t*-test.

We further explored the effect of MitoTEMPO on inflammatory response in the liver of HFD mice. The mRNA level of the chemokine *MCP-1*, which may mediate the trafficking of MDSCs from circulation to liver tissues and induce chronic inflammation, was increased by 6.8-folds in the HFD group than in the lean group (*P* < 0.05) (Figure 4(a)). However, treatment HFD mice with MitoTEMPO caused about a 3-fold decrease in *MCP-1* mRNA expression (*P* < 0.05) (Figure 4(a)). Moreover, MCP-1 protein expression showed a 3-fold increase in the HFD group compared with the lean group (*P* < 0.01) and a 1.4-fold decrease in the HFD+Mito group (*P* < 0.05) (Figure 4(b)). The mRNA level of *C-C chemokine receptor 2* (*CCR2*), the receptor of *MCP-1*, increased by 2.3-folds in the HFD group (*P* < 0.05) and decreased by 2.5-folds after MitoTEMPO treatment (*P* < 0.05) (Figure S1).

The mRNA levels of *S100A8* and *S100A9*, two MDSC-associated proinflammatory cytokines, were increased about 2.5-folds and 2.3-folds in the HFD group compared with the lean group (*P* < 0.01) but dropped 5.9-folds and 5.2-folds after MitoTEMPO administration (*P* < 0.001) (Figures 4(c) and 4(d)), respectively. Similarly, the protein levels of S100A8 and S100A9 were also increased about 3.1-folds and 1.6-folds in the HFD group compared with the lean group (*P* < 0.001) and decreased 1.9-folds and 1.4-folds after MitoTEMPO administration (*P* < 0.01) (Figures 4(e) and 4(f)), respectively.

### 3.5. MitoTEMPO Suppressed the Expression of Liver Fibrosis-Associated Genes

**Figure 5:**
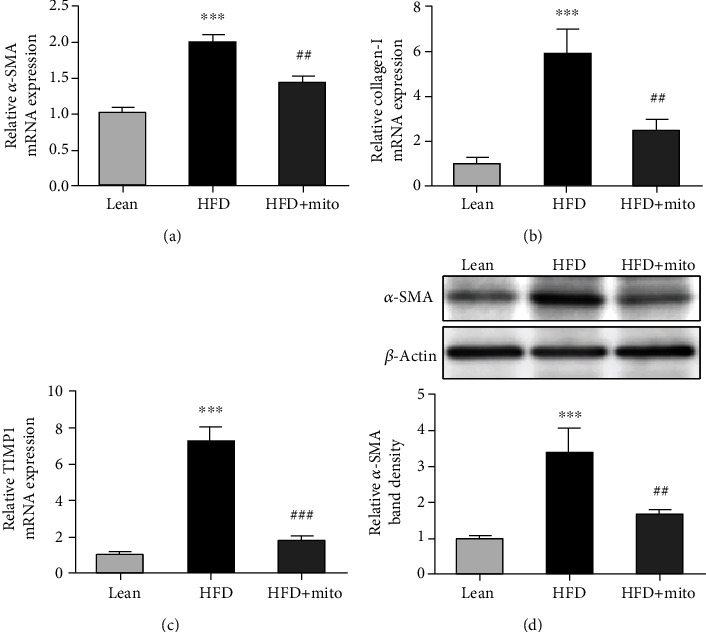
Effects of MitoTEMPO on HFD-induced liver fibrosis. (a–c) The mRNA levels of *α-SMA* (a), *Collagen-I* (b), and *TIMP1* (c) in the liver tissues of the lean, HFD, and HFD+Mito groups. mRNA levels were normalized to *GAPDH* expression and shown as fold change (2^-*ΔΔ*Ct^). (d) The protein expression of *α*-SMA in the liver tissues of each group. The bottom shows the relative band intensity of *α*-SMA to *β*-actin. Data represent the mean ± SEM from three independent experiments. HFD vs. lean: ^∗∗∗^*P* < 0.001, HFD+Mito vs. HFD: ^##^*P* < 0.01, ^###^*P* < 0.001, unpaired *t*-test.

Finally, we examined the different expressions of fibrosis-associated genes among these groups. The mRNA levels of *α-SMA*, *Collagen-I*, and *TIMP1* were moderately elevated in the liver of the HFD group compared with the lean group (*P* < 0.001) (Figures 5(a)–5(c)). Decreased mRNA levels of these genes were detected in the liver of the HFD group after MitoTEMPO treatment (*P* < 0.01) (Figures 5(a)–5(c)). The protein level of *α*-SMA was also increased in the HFD group (*P* < 0.001) and decreased after MitoTEMPO treatment (*P* < 0.01) (Figure 5(d)).

## 4. Discussion

In this study, we used a HFD-induced obese mouse model, which led to NAFLD-associated consequences, such as hepatic steatosis, chronic inflammation, and fibrosis [23]. We found an increased rate of CD11b^+^Gr-1^+^ MDSCs in the peripheral circulation of HFD-fed mice. Besides, the expression of chemokine MCP-1, which may mediate MDSC trafficking from peripheral blood to the liver [24, 25], was increased in the liver of HFD mice. Indeed, as we hypothesized, we found an increased accumulation of Gr-1^+^ cells in the liver of HFD-induced obese mice, which may further contribute to the immune dysregulation and chronic inflammatory response. The expressions of proinflammatory cytokines S100A8 and S100A9, which are secreted by a variety of cells including MDSCs [26], were also upregulated in the liver of obese mice. MitoTEMPO appeared to ameliorate obesity-induced NAFLD-associated symptoms via suppressing MDSC accumulation and inhibiting chronic inflammatory response.

Accumulating evidence has demonstrated that the development of NAFLD is related to adaptive immune responses. It is reported that in the formation of nonalcoholic fatty liver, Th17 cells were increased in the liver, and the Th17/resting Treg ratio was elevated both in the peripheral blood and the liver [27]. Sutti et al. observed increased hepatic CD4^+^ cells in a methionine-choline-deficient (MCD) diet-induced NASH mouse model [28]. However, a recent report pointed out that in NAFLD, the intrahepatic tumor-suppressive CD4^+^ T cells were selectively damaged, leading to the disorder of lipid metabolism [16]. The opposite results may be due to different methods for NAFLD model establishment. In this study, we established a NAFLD model using high fat diet-induced mice, which is more similar to the pathogenesis in humans.

MDSCs are a group of heterogeneous immune cells derived from the bone marrow with immunosuppressive function [29]. In mice, MDSCs are characterized with the expressions of cell surface markers CD11b and Gr-1 [30]. To date, studies on the phenotypes and functions of MDSCs in obesity-induced NAFLD are few [31, 32]. A previous study showed mice fed a high fat diet caused accumulation of monocytic-like MDSCs in the liver [33]. Our previous study showed in the peripheral circulation of obese individuals that the frequency of monocytic-like MDSCs was increased compared with that of healthy individuals [34]. In our present study, HFD induced an increased frequency of CD11b^+^Gr-1^+^ MDSCs in peripheral circulation and an accumulation of Gr-1^+^ cells in the liver.

It is reported that dysregulation of MDSCs is relevant with chronic inflammation [35]. A high level of proinflammatory chemokine MCP-1 and numbers of CCR2^+^ (MCP-1 receptor) MDSCs were present in obese mice bearing renal tumor [36]. In this study, we found increased expressions of MCP-1 and CCR2 in the liver of obese mice, which may accelerate the accumulation of MDSCs in the liver. In addition, the expressions of proinflammatory cytokines S100A8 and S100A9, which were considered as markers of human monocytic MDSCs [37], were increased in the liver tissues of obese mice as well. Moreover, S100A8 and S100A9 are not just markers of MDSCs; they can also be detected in neutrophils, monocytes, and early differentiation stages of macrophages [38–40]. Thus, the accumulated MDSCs in the liver exert immunological functions in part by regulating local inflammation. The proinflammatory S100A8 and S100A9 proteins can regulate the accumulation of MDSCs, and in turn, MDSCs can also synthesize and secrete S100A8 and S100A9 proteins [27, 41]. Our data suggested that the increased S100A8 and S100A9 derived from MDSCs may in turn exacerbate chronic inflammatory reaction in the liver during NAFLD progression.

There are a few studies of MitoTEMPO, a mitochondrial antioxidant, in NAFLD. It has been reported that MitoTEMPO prevented lipopolysaccharide- (LPS-) induced inflammatory and liver injury in rats [42]. MitoTEMPO delays NAFLD-promoted HCC in an MCD diet-induced mouse NAFLD model [16]. Treatment with MitoTEMPO prevented insulin resistance and diastolic dysfunction in a HFD-induced diabetic mouse model [43]. The mechanism by which MitoTEMPO functions in these diseases is mostly via regulating oxidative stress. However, whether MitoTEMPO is involved in the regulation of immune activities in HFD-induced NAFLD has not been clarified. On the basis of our studies, we found MitoTEMPO treatment reduced infiltration of local inflammatory cells and alleviated hepatic steatosis in the liver of the HFD mice. These changes by MitoTEMPO may partially depend on reducing the local immunocyte accumulation and suppressing MDSC-associated proinflammatory factors in the liver, suggesting that MitoTEMPO may ameliorate NAFLD at least in part by regulating MDSC functions. However, whether MitoTEMPO directly or indirectly regulates MDSCs has not been clarified.

Extracellular matrix (ECM) proteins are generally produced by activated hepatic stellate cells (HSCs). The activation of HSCs is implicated in hepatic fibrogenesis by producing profibrogenic factors and by remodeling the ECM [44]. Our results showed that MitoTEMPO suppressed fibrosis-associated genes such as *α*-SMA, Collagen-I, and TIMP1 in HFD mice, indicating MitoTEMPO may have antifibrosis function.

## 5. Conclusion

In conclusion, our data suggest that dysregulation of immune activities exacerbates high fat diet-induced NAFLD. Antioxidant MitoTEMPO may provide novel pharmacological functions on immune and inflammation regulation in obese-induced NAFLD.

## Figures and Tables

**Table 1:** Primer sequences for quantitative PCR.

Gene	Forward sequence	Reverse sequence
*MCP-1*	CACAACCACCTCAAGCAC	AAGGGAATACCATAACATCA
*S100A8*	CTTCAAGACATCGTTTGAAAGG	ATTCTTGTAGAGGGCATGGT
*S100A9*	TTAGCCTTGAAGAGCAAGAAGATGG	AGCTCAGCTGATTGTCCTGGT
*α-SMA*	ACTGGGACGACATGGAAAAG	GTTCAGTGGTGCCTCTGTCA
*Collagen-I*	TGGCCTTGGAGGAAACTTTG	CTTGGAAACCTTGTGGACCAG
*TIMP1*	GATATGCCCACAAGTCCCAGAACC	GCACACCCCACAGCCAGCACTAT
*GAPDH*	AACAGGGTGGTGGACCTCAT	GGGATAGGGCCTCTCTTGCT

## Data Availability

The data used to support the findings of this study are available from the corresponding author upon request.

## Supplementary Materials

Supplementary Materials Figure S1: effect of MitoTEMPO on the mRNA expression of CCR2 in the liver tissues of mice. qRT-PCR assay was conducted to measure the CCR2 mRNA level. Data are represented as the mean ± SEM. HFD vs. lean: ^∗^*P* < 0.05, HFD+Mito vs. HFD: ^#^*P* < 0.05, unpaired *t*-test.
