# Supplementary material for: Protective Effects of MitoTEMPO on Nonalcoholic Fatty Liver Disease via Regulating Myeloid-Derived Suppressor Cells and Inflammation in Mice
Source: Biomed Res Int. 2020 Jul 30;2020:9329427. doi: 10.1155/2020/9329427 (PMC7414374; doi:10.1155/2020/9329427)
Supplement: Supplementary Materials — Figure S1: effect of MitoTEMPO on the mRNA expression of CCR2 in the liver tissues of mice. qRT-PCR assay was conducted to measure the CCR2 mRNA level. Data are represented as the mean ± SEM. HFD vs. lean: ∗P < 0.05, HFD+Mito vs. HFD: #P < 0.05, unpaired t-test. [file 9329427.f1.doc]

**Supplementary materials**

**
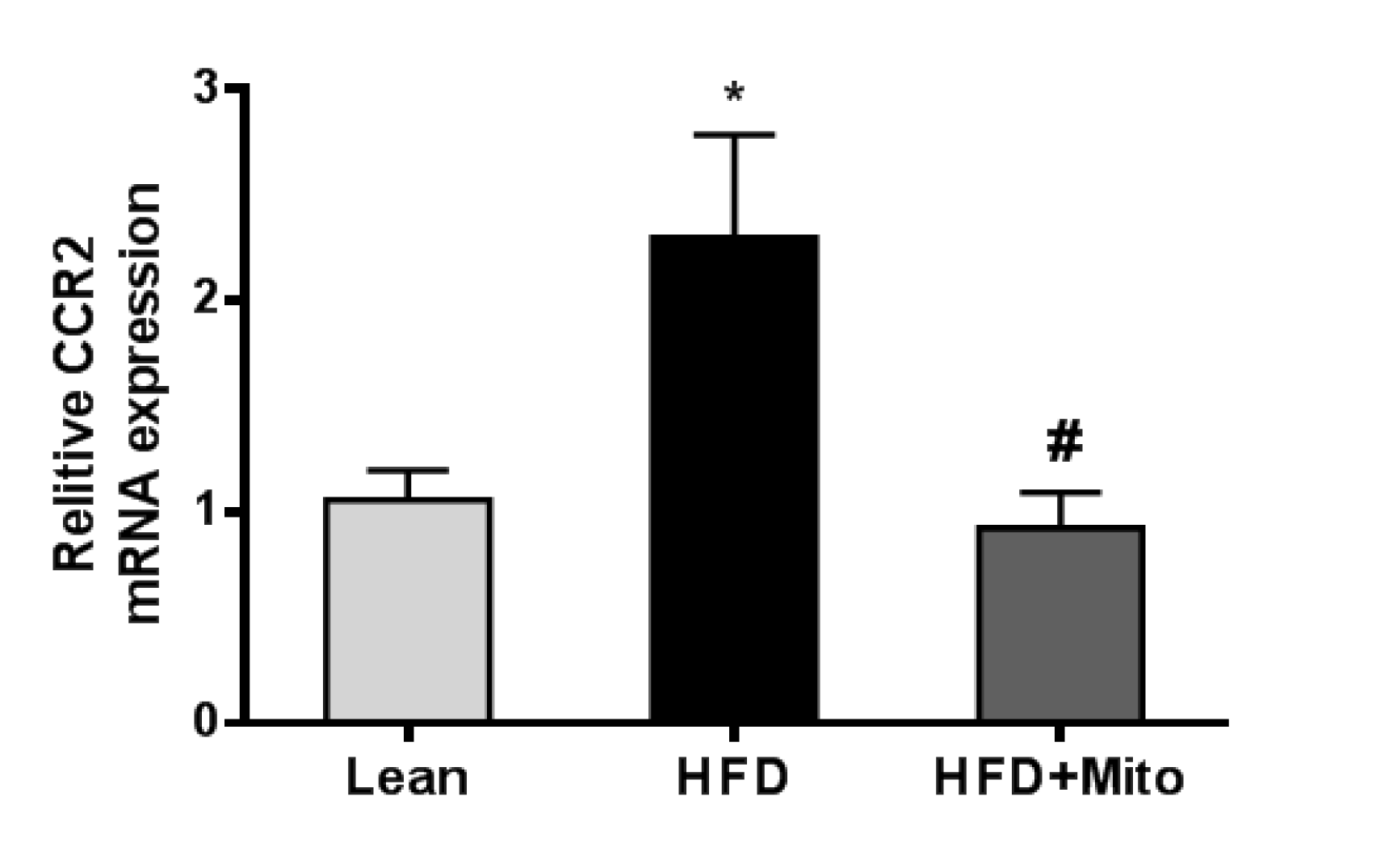
**

**Figure S1** Effect of MitoTEMPO on the mRNA expression of *CCR2* in the liver tissues of mice. qRT-PCR assay was conducted to measure CCR2 mRNA level. Data are represented as the mean ± SEM. HFD vs. Lean: *, *P* < 0.05, HFD + Mito vs. HFD: #, *P* < 0.05, unpaired *t*-test.
